# Divergent scaling of respiration rates to nitrogen and phosphorus across four woody seedlings between different growing seasons

**DOI:** 10.1002/ece3.3419

**Published:** 2017-09-18

**Authors:** Ruirui Fan, Jun Sun, Fuchun Yang, Man Li, Yuan Zheng, Quanlin Zhong, Dongliang Cheng

**Affiliations:** ^1^ Fujian Provincial Key Laboratory of Plant Ecophysiology Fujian Normal University Fuzhou Fujian China; ^2^ Key Laboratory of Humid Subtropical Eco‐geographical Process Ministry of Education Fuzhou Fujian China

**Keywords:** allometry, growing and dormant seasons, metabolic scaling theory, nitrogen, phosphorus, respiration rates

## Abstract

Empirical studies indicate that the exponents governing the scaling of plant respiration rates (*R*) with respect to biomass (*M*) numerically vary between three‐fourth for adult plants and 1.0 for seedlings and saplings and are affected by nitrogen (N) and phosphorus (P) content. However, whether the scaling of *R* with respect to *M* (or N and P) varies among different phylogenetic groups (e.g., gymnosperms vs. angiosperms) or during the growing and dormant seasons remains unclear. We measured the whole‐plant *R* and *M*, and N and P content of the seedlings of four woody species during the growing season (early October) and the dormant season (January). The data show that (i) the scaling exponents of *R* versus *M*,* R* versus N, and *R* versus P differed significantly among the four species, but (ii), not between the growing and dormant seasons for each of the four species, although (iii) the normalization constants governing the scaling relationships were numerically greater for the growing season compared to the dormant season. In addition, (iv) the scaling exponents of *R* versus *M*,* R* versus N, and *R* versus P were numerically larger for the two angiosperm species compared to those of the two gymnosperm species, (v) the interspecific scaling exponents for the four species were greater during the growing season than in the dormant season, and (vi), interspecifically, P scaled nearly isometric with N content. Those findings indicate that the metabolic scaling relationships among *R*,* M*, N, and P manifest seasonal variation and differ between angiosperm and gymnosperm species, that is, there is no single, canonical scaling exponent for the seedlings of woody species.

## 1 INTRODUCTION

Respiration consumes approximately 50% of the total CO_2_ fixed by photosynthesis (Ryan, 1991). It therefore is a major factor influencing the energy budget of individual plants as well as the energy budget of forested ecosystems (Demesin, 2003; Paembonan, Hagihara, & Hozumi, 1991). Research has shown that respiration rates can be related to a plant's standing biomass via the general allometric equation *R* = β*M* ^α^, where *R* denotes respiration rate, *M* is biomass, β is a normalization constant, and α is a scaling exponent. Based on the assumption that all organisms have a fractal network structure, the metabolic scaling theory predicts that *R* scales as the three‐fourth power of *M* across a broad range of diverse plant species (West, Brown, & Enquist, 1997, 1999). However, Reich, Tjoelker, Machado, and Oleksyn (2006) have shown that whole‐plant respiration rates scale nearly isometrically with *M* (i.e., α = 1.0) for seedlings and very small juvenile plants, and Enquist et al. (2007) report that scaling exponents of *R* = βM^α^ are close to 1.0 for seedlings and shift to three‐fourth as plants increase in overall size. Several empirical and theoretical studies have supported the ontogenetic shifting of the metabolic scaling exponent and argue that the possible reason of this shifting is biomass allocation to photosynthetic tissues versus nonphotosynthetic tissues such as secondary xylem (Cheng, Li, Zhong, & Wang, 2010; Mori et al., 2010; Niklas, 1994; Niklas & Enquist, 2002; Peng, Niklas, Reich, & Sun, 2010). Nonetheless, the factors responsible for this shift in the numerical values of α remains poorly understood.

One possible explanation for this shift is physiological changes in the allocation or utilization of critical nutrients, such as nitrogen (N) and phosphorus (P) (Niklas, Owens, Reich, & Cobb, 2005). N is a chief component of key enzymes involved in plant metabolic processes (Atkinson, Hellicar, Fitter, & Atkin, 2007; Machado & Reich, 2006; Reich et al., 2010). Consequently, respiration rates are limited by N availability for plants of different life forms and phylogenetic groups (Reich, Walters, Tjoelker, Vanderklein, & Buschena, 1998; Ryan, Hubbard, Pongracic, Raison, & Mcmurtrie, 1996). However, there is no consensus on the scaling of *R* with respect to N. Reich et al. (2006) have reported that whole‐plant respiration rates isometrically scale to total N content, whereas Wang, Huang, Deng, and Liu (2015) report that the scaling exponents of *R* with respect to N in seedlings for herbaceous and woody deciduous species are significantly lower than 1.0. Likewise, P is another essential component of nucleic acids and many proteins, including enzymes involved in the respiratory release of energy contained in sugars and the regulation of various metabolic pathways (Theodorou & Plaxton, 1993; Wang et al., 2015; Wright et al., 2004). Thus, P might also be a good predictor of respiration rates (Elser, Fagan, Kerkhoff, Swenson, & Enquist, 2010; Elser et al., 2000; Hedin, 2006; Sterner & Elser, 2002).

Nevertheless, the scaling relationships of *R* with respect to *M*, N, and P are likely to differ among different species groups, and they may vary as a function of climatic and soil conditions (Enquist et al., 2007; McCarthy & Enquist, 2007; Price & Enquist, 2007; Sperry et al., 2012; Von Allmen et al., 2012). For example, studies have shown that scaling relationships are sensitive to plant phylogenetic groups (angiosperms vs. gymnosperms) (Glazier, 2013), Cheng, Niklas, Zhong, Yang, and Zhang (2014) report that the scaling exponents of angiosperms are numerically larger than those of gymnosperms across seedlings from different species, and Bond (1989) concludes that the growth rate of gymnosperms is lower than that of angiosperms. Considering that growth rates are a proxy indicator of respiration rates (Brown, Gillooly, Allen, Savage, & West, 2004; West et al., 1999), it is likely that the scaling exponents of *R* with respect to *M*, N, and P will differ between angiosperms and gymnosperms. However, studies focusing on these scaling relationships remain scarce.

It is also reasonable to surmise that these scaling relationships might also change over the course of the growing season and during the dormant period. The classical plant physiological model proposed by McCree and Šetliik (1970)states that plant respiration rates consist of growth and maintenance respiration. The former is predicted to be proportional to growth rate, whereas maintenance respiration is predicted to be proportional to standing living biomass. Given that growth rates of larger plant are predicted to scale as the ¾ power of the biomass (Niklas, 2006; West et al., 1999), McCree's plant physiological model implies that the scaling exponents of *R* with respect to *M* for large plants should shift from 1.0 to ¾ depending on the relative proportions of growth or maintenance respiration to total respiration. Specifically, if the maintenance respiration rate is lower than growth respiration during the growing season, the scaling exponent of *R* with respect to *M* should be close to ¾. In contrast, if growth respiration is constrained by cool temperatures during the dormant season, the metabolic scaling should be close to 1.0. In fact, Hoque, Sharma, Suwa, Mori, and Hagihara (2010) report that the scaling exponent of *R* with respect to *M* for adult trees is close to 1.0 in the dormant season, but close to ¾ in the growing season.

In order to further explore the complex relationships among *R*,* M*, N, P, and rendering the scaling relationship between *R* and N (as well as P) content of seedlings in divergent growth seasons unclear, we measured the whole‐plant *R*,* M*, N, and P content for the seedlings of four woody species during the growing season (early October) and during the dormant season (January), and we compared the numerical values of the scaling exponents governing these relationships to the expectations of the metabolic scaling theory.

## 2 MATERIALS AND METHODS

### 2.1 Plant material and experimental conditions

The experiment was conducted at the Forestry Science and Technology Promotion Center in Shunchang County, Fujian Province, China (26°46′N, 117°52′E). The mean annual temperature is 18.5°C, with an average of 26.85°C in the warmest month (July) and 9.1°C in the coldest month (January). The mean annual precipitation is 1756 mm, and the major soil texture is mainly sandy clay loam. The basic morphometrics of the two gymnosperm species (*Cunninghamia lanceolata* (Lamb.) Hook. and *Pinus massoniana* Lamb.) and the two angiosperm species (*Machilus pauhoi* Kanehira and *Phoebe bournei* (Hemsl.) Yang) are provided in Appendix S1, they are the typical forest species found in the subtropical monsoon climate of the Fujian Province.

Seeds were disinfected using a 5% KMnO_4_ solution for 30 min and then rinsed thoroughly in distilled water at 20°C for 24 hr before sowing. The seeds were planted in wet sand and placed in a growth chamber until they germinated. Subsequently, they were planted individually in circular plastic containers filled with decomposed sawdust (in March of 2012). The seedlings were cultivated outdoors under sunshade nets without fertilization. The shelter had no sidewalls, so the air temperature, wind speed, and relative humidity were similar to ambient conditions.

Under the foregoing conditions, the plants had two growth peaks (i.e., from March to June, and from September to November). Accordingly, we measured respiration during the growing season (in early October, 2013) and during the dormant season (in January, 2014). Thirty healthy seedlings of each species were collected during each of the two seasons, yielding a total of 240 individuals, ranging in fresh weight from 1.1 to 50.5 g. After harvesting, whole plants were immediately transported to the laboratory for respiration measurements.

### 2.2 Dark respiration measurements

Whole‐plant dark respiration rates were measured using the protocols of Peng et al. (2010). Briefly, whole plants were harvested to minimize fine root loss, the roots were immediately immersed in water, plants were placed in darkness for 2 hr, and respiration was measured thereafter. A soil respiration chamber with a CIRAS‐2 Portable Photosynthesis System (PP System) was used to measure whole‐plant respiration rates for small seedlings. A customized chamber (2 L) was used to measure whole‐plant respirations rates for larger plants (Peng et al., 2010). The chambers were sealed with a thin layer of petroleum jelly to minimize CO_2_ leakage after plants were placed in the chambers. To account for the effects of temperature on dark respiration rates, measured respiration rates were adjusted to those rates corresponding at a standardized temperature (i.e., 24°C) using a previously published temperature‐dependent *Q*_10_ model (Atkin & Tjoelker, 2003).

### 2.3 Biomass measurements

After respiration measurements were taken, each seedling was cut at the base of the stem to separate the above‐ground from the below‐ground part (roots) of each plant, followed by separation of the above‐ground portion into leaf and stem. All leaf, stem, and root parts were dried at 65°C for 72 hr to measure dry weight.

### 2.4 Nitrogen and phosphorus measurements

After being weighed, samples were ground to a powder with a grinder, which was then passed through a 100 mesh sieve (0.15 mm). The N content was determined with an Element Analyzer (VARIO EL III Element Analyzer, Elementar, Germany). The P content was measured using the molydate/ascorbic acid method and a continuous flow analyzer (SKALAR SAN++, Netherlands) after H_2_SO_4_–H_2_O_2_ digestion.

### 2.5 Published data sources

Data for 500 laboratory and field‐grown plants spanning 43 species under four experimental conditions were taken from Reich et al. (2006), and data for 150 laboratory and field‐grown seedlings representing 30 herbaceous species and 20 woody deciduous species were taken from Wang et al. (2015).

### 2.6 Statistical analysis

Data of *R*,* M*, N, and P were log_10_‐transformed to generate a normal distribution. Model Type II regression was used to determine the scaling exponent and the normalization constant of log–log linear relationships (i.e., α and log β, respectively) using the (Standardized) Major Axis Estimation package “smatr” version 3.4‐3 in R software (R Development Core Team 2013; Warton, Duursma, Falster, & Taskinen, 2012). This package was also used to determine whether the numerical values of α for *R* with respect to *M*, N, and P differed among the four species and to provide the Model Type II equivalent of OLS standard analyses of covariance (Warton, Wright, Falster, & Westoby, 2006; Warton et al., 2012). The significance level for scaling exponent heterogeneity was *p *<* *.05 (i.e., scaling exponent heterogeneity was rejected if *p *>* *.05). The common scaling exponent estimate is the slope estimate obtained from a pooled variance/covariance matrix. For several bivariate sets of observations, this function tests if the line‐of‐best‐fit has a common slope for all observations, when the line‐of‐best‐fit is estimated using the major axis, standardized major axis, or a more general version of these methods in which the error variance ratio is estimated from the data.

## 3 RESULTS

### 3.1 The scaling relationships between respiration rates and biomass

**Figure 1:**
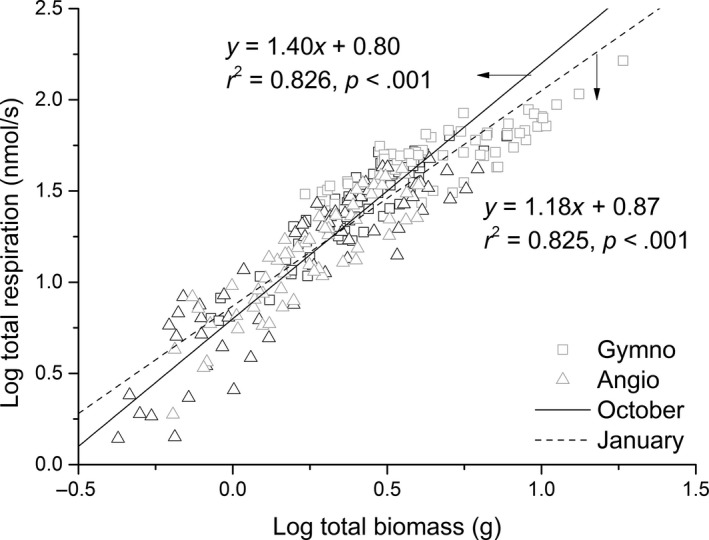
Log–log bivariate plot for seedlings respiration rates versus biomass for gymnosperm and angiosperm species during the growing and dormant seasons (in October and January)

The scaling exponents of *R* versus *M* differed significantly among the four species (*p *= .023 and *p *= .009 for the growing and dormant season, respectively; Table 1). However, for each of the four species, the scaling exponent of *R* versus *M* did not significantly differ between the two seasons and thus shared a common scaling exponent (Table 2). In contrast, the normalization constants for the *R* versus *M* relationship for each species differed significantly between the two seasons (*p *<* *.001; Table 1). The scaling exponent of *R* versus *M* was significantly larger than that of the gymnosperm species when the data from each of the two species groups were pooled from the two seasons (Table 1, Figure 1). Analyses also indicated that, across all four species, the interspecific scaling exponent of *R* versus *M* was significantly larger for the growing season compared to the dormant season (*p *<* *.001; Table 1, Figure 1).

**Table 1:** Summary of regression parameters (scaling exponents and normalization constants; α and log β, respectively) for *R* versus *M* relationships of woody species during the growing and dormant seasons

Species	*n*	October			January		
		α (95% CI)	log β (95% CI)	*r* ^2^	α (95% CI)	log β (95% CI)	*r* ^2^
*Cunninghamia lanceolata*	30	1.09 (0.92, 1.30)	1.04 (0.95, 1.13)	.794	1.18 (1.01, 1.39)	0.73 (0.56, 0.90)	.827
*Pinus massoniana*	30	1.09 (0.98, 1.20)	0.88 (0.84, 0.93)	.932	0.93 (0.81, 1.07)	1.18 (1.12, 1.25)	.868
*Machilus pauhoi*	30	1.37 (1.22, 1.53)	0.64 (0.57, 0.71)	.912	1.30 (1.14, 1.48)	0.90 (0.84, 0.97)	.885
*Phoebe bournei*	30	1.16 (1.03, 1.30)	0.97 (0.93, 1.02)	.911	1.13 (0.95, 1.34)	0.76 (0.71, 0.82)	.793
*Gymnosperms*	60	1.29 (1.16, 1.44)	0.88 (0.82, 0.94)	.835	0.68 (0.58, 0.79)	1.24 (1.17, 1.32)	.671
*Angiosperms*	60	1.38 (1.23, 1.55)	0.78 (0.71, 0.84)	.802	1.43 (1.27, 1.61)	0.79 (0.73, 0.84)	.799
*All*	120	1.40 (1.30, 1.51)	0.80 (0.76, 0.85)	.826	1.18 (1.10, 1.28)	0.87 (0.82, 0.92)	.825

All regressions are statistically significant (*p *<* *.001).

95% CI: The 95% confidence interval.

**Table 2:** Summary of common scaling exponents for *R* versus *M*,* R* versus N*,* and *R* versus P for four species between the growing and dormant seasons

Species	*R* versus *M*		*R* versus N		*R* versus P	
	CSE (95% CI)	*p*	CSE (95% CI)	*p*	CSE (95% CI)	*p*
*Cunninghamia lanceolata*	1.14 (1.01, 1.28)	.496	1.21 (1.07, 1.37)	.149	1.24 (1.05, 1.46)	.504
*Pinus massoniana*	1.03 (0.95, 1.12)	.083	1.07 (0.98, 1.17)	.081	0.94 (0.85, 1.04)	.521
*Machilus pauhoi*	1.34 (1.23, 1.46)	.587	–	–	1.52 (1.37, 1.68)	.060
*Phoebe bournei*	1.15 (1.04, 1.26)	.801	1.21 (1.10, 1.33)	.484	1.30 (1.17, 1.44)	.248

CSE: Common scaling exponent.

### 3.2 The scaling relationships between respiration rates and nitrogen

**Figure 2:**
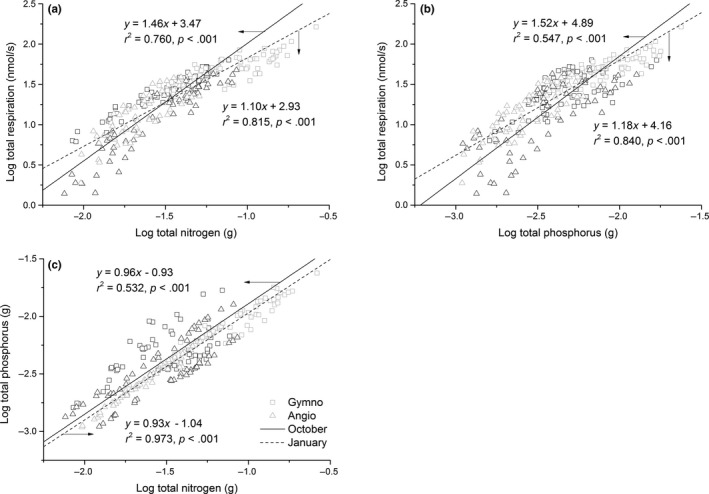
Log–log bivariate plot for seedlings respiration rates versus nitrogen (a) and phosphorus (b), and phosphorus to nitrogen (c) for gymnosperm and angiosperm species during the growing and dormant seasons (in October and January)

With one exception, the numerical value of the scaling exponent of *R* versus N varied significantly among the four species between the two seasons (*p *<* *.001 for growing season and *p *=* *.013 for the dormant season; Table 3). The exception was *M. pauhoi*. For this species, the scaling exponents were not significantly different between the two seasons (Table 2). When data were divided into the two species groups and pooled, the scaling exponents of *R* versus N for the two angiosperms were significantly larger than those of the two gymnosperms in both seasons (*p *< .001; Table 3, Figure 2a). Across the entire data set, the scaling exponent of *R* versus N was significantly larger in the growing season than that in the dormant season (*p *< .001; Table 3, Figure 2a).

**Table 3:** Summary of regression parameters (scaling exponents and normalization constants; α and log β, respectively) for *R* versus N relationships for four species during the growing and dormant seasons

Species	*n*	October			January		
		α (95% CI)	log β (95% CI)	*r* ^2^	α (95% CI)	log β (95% CI)	*r* ^2^
*Cunninghamia lanceolata*	30	1.13 (0.97, 1.32)	3.08 (2.84, 3.32)	.841	1.35 (1.11, 1.64)	3.01 (2.77, 3.25)	.744
*Pinus massoniana*	30	1.13 (1.02, 1.25)	3.14 (2.94, 3.33)	.929	0.96 (0.83, 1.12)	2.88 (2.69, 3.07)	.847
*Machilus pauhoi*	30	1.64 (1.47, 1.83)	3.57 (3.28, 3.87)	.918	1.28 (1.12, 1.47)	3.38 (3.09, 3.66)	.872
*Phoebe bournei*	30	1.18 (1.06, 1.32)	2.98 (2.98, 3.17)	.917	1.27 (1.07, 1.50)	3.09 (2.72, 3.45)	.807
*Gymnosperms*	60	1.04 (0.98, 1.11)	2.98 (2.88, 3.08)	.939	0.72 (0.61, 0.84)	2.50 (2.37, 2.63)	.631
*Angiosperms*	60	1.52 (1.39, 1.65)	3.42 (3.22, 3.62)	.896	1.63 (1.41, 1.88)	3.81 (3.43, 4.20)	.701
All	120	1.46 (1.34, 1.60)	3.47 (3.27, 3.68)	.760	1.10 (1.02, 1.19)	2.93 (2.81, 3.05)	.815

All regressions are statistically significant (*p *<* *.001).

### 3.3 The scaling relationship between respiration rates and phosphorus

Significant differences in the numerical values of the scaling exponents were observed for the *R* versus P relationship among the four species in both the growing season and the dormant season (*p *< .001 for growing season and *p *< .05 for the dormant season; Table 4). In contrast, the scaling exponents for each of the four species were not significantly different between the two seasons, nor were there differences in the numerical values of the normalization constants (*p *< .001; Table 2). The scaling exponents also differed significantly between two different species groups; those of the angiosperms was larger than those of the gymnosperms for both of the two seasons (*p *< .001; Table 4, Figure 2b). Furthermore, across all four species, the scaling exponent of *R* versus P in the growing season was larger than that in the dormant season (*p* < .001; Table 4, Figure 2b). Our analyses also indicated that P scaled nearly isometrically with N in both seasons (Table 4, Figure 2c).

**Table 4:** Summary of regression parameters (scaling exponents and normalization constants; α and log β, respectively) for *R* versus P relationships for four species during the growing and dormant

Species	*n*	October			January		
		α (95% CI)	log β (95% CI)	*r* ^2^	α (95% CI)	log β (95% CI)	*r* ^2^
*Cunninghamia lanceolata*	30	1.31 (1.03, 1.67)	4.63 (3.89, 5.38)	.608	1.18 (0.94, 1.47)	4.05 (3.53, 4.56)	.660
*Pinus massoniana*	30	0.92 (0.81, 1.04)	3.37 (3.11, 3.64)	.897	0.98 (0.83, 1.17)	3.80 (3.43, 4.17)	.803
*Machilus pauhoi*	30	1.63 (1.45, 1.84)	4.85 (4.38, 5.32)	.905	1.36 (1.17, 1.58)	4.77 (4.25, 5.28)	.851
*Phoebe bournei*	30	1.35 (1.20, 1.53)	4.69 (4.27, 5.11)	.901	1.19 (1.00, 1.42)	4.07 (3.52, 4.62)	.794
*Gymnosperms*	60	1.23 (1.01, 1.50)	4.27 (3.69, 4.84)	.424	0.86 (0.75, 0.99)	3.48 (3.23, 3.73)	.708
*Angiosperms*	60	1.55 (1.31, 1.85)	4.94 (4.26, 5.61)	.556	1.61 (1.38, 1.87)	5.27 (4.65, 5.90)	.670
All	120	1.52 (1.34, 1.71)	4.89 (4.44, 5.34)	.547	1.18 (1.10, 1.27)	4.16 (3.96, 4.36)	.840

All regressions are statistically significant (*p *< .001).

## 4 DISCUSSION

### 4.1 Variation of respiration scaling in different phylogenetic groups

Our results reveal that the numerical values of the scaling exponents for the *R* versus *M*,* R* versus N, and *R* versus P scaling relationships are higher for angiosperms than those of gymnosperms in both the growing and dormant seasons. These results indicate that angiosperm seedlings can have higher respiration rates compared to gymnosperms. These differences likely reflect differences in the functional traits of the two species groups and may help to explain why angiosperms tend to have higher growth rates than most gymnosperms. For example, Lusk (2011) concludes that gymnosperm trees tend to have longer‐lived leaves characterized by a greater mass per area (LMA) and lower mass‐based photosynthetic capacity compared to angiosperm trees. LMA is an important trait that negatively correlates with variations in the growth rates of different species (Poorter, Niinemets, Poorter, Wright, & Villar, 2009; Wright et al., 2004), it is reasonable to speculate that gymnosperms would, on average, have lower growth rates (and thus lower respiration rates) compared to angiosperms. Indeed, this is consistent with the “seedling hypothesis” for angiosperm dominance proposed by several authors (Bond, 1989; Cornelissen, Diez, & Hunt, 1996; Enright, Bartlett, & Defreitas, 1993; Read, 1995; Reich, Tjoelker, Walters, Vanderklein, & Buschena, 1998), which in turn is consistent with the observation that gymnosperms tend to have lower growth rates than angiosperms (Lusk & Matus, 2000).

### 4.2 Variation of respiration versus mass during the growing seasons and dormant seasons

The scaling exponents of *R* versus *M* are numerically larger in the growing season compared to the dormant season across the four woody species examined during the course of this study, whereas Hoque et al. (2010) state that the scaling exponents for *R* versus *M* are approximately one, larger for mangroves during the dormant season, and decrease to nearly ¾ during the growing season. These discrepancies require an explanation, which might emerge from differences in the relative proportions of maintenance and growth respiration for seedlings compared to larger plants during the growing and dormant seasons. For example, the physiological model proposed by McCree and Šetliik (1970) states that growth respiration is proportional to growth rate and that maintenance respiration is directly proportional to biomass. Given that the growth rate of larger plants is expected to scale as the ¾ of standing biomass (West et al., 1999), the metabolic scaling for larger plants is predicted to be close to ¾ when the maintenance respiration rate is lower than the growth respiration during the growing season, and close to one when growth respiration is less than maintenance respiration during the dormant season (i.e., the scaling exponent for *R* vs. *M* will shift from close to 1.0 to ¾ as plants grow and increase in size). Indeed, this prediction is consistent with the findings of Hoque et al. (2010).

However, for small plants, the metabolic scaling theory predicts that growth rates should scale one‐to‐one (isometrically) with standing biomass (West et al., 1997), whereas our results indicate that the scaling exponents of *R* versus *M* are significantly larger than 1.0 (i.e., α = 1.40) during the growing season, and approximately isometric during the dormant season (i.e., α = 1.18) for each of the four species examined in this study. Therefore, our results provide only partially support the physiological model of respiration provided by McCree and Šetliik (1970).

### 4.3 Scaling relationships between respiration and nitrogen and phosphorus

**Figure 3:**
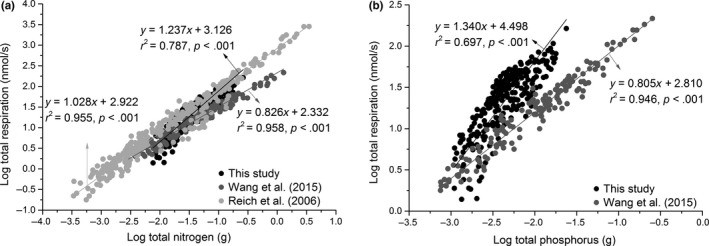
Comparison of respiration rates to nitrogen (a) and phosphorus (b) for Reich et al. (2006) and Wang et al. (2015)

Our results indicate that the interspecific scaling exponent for the *R* versus N scaling relationship is significantly larger than 1.0 (i.e., α = 1.46) during the growing season and approximately isometric during the dormant season (i.e., α = 1.10) across the four species. Furthermore, when the entire data are pooled for both seasons, *R* scaled as the 1.24‐power of N content, which is statistically significantly >1.0 (Figure 3a). These results are consistent with the 1.14–1.60 scaling of *R* versus N reported by Peng et al. (2010), but they are inconsistent with the nearly isometric scaling relationship (i.e., α = 1.0) of *R* versus N reported by Reich et al. (2006) and the results reported by Wang et al. (2015) (Figure 3a). The difference between our results and those reported by Reich et al. (2006) and Wang et al. (2015) most likely emerges from the positive correlation between N content on respiration rates. Because our seedling had a higher N content a positive correlation between N content and *R*, we observed numerically larger scaling exponents for *R* versus N (Figure 3a) compared to those reported by other studies.

We found that the scaling exponent for *R* versus P is numerically similar to that of *R* versus N when the data were pooled (Figure 3b). This observation is not surprising because the uptake, transport, and allocation mechanisms for P and N are very similar in plants (Feild & Brodribb, 2001; Jeschke, Kirkby, Peuke, Pate, & Hartung, 1997; Kilgore, Patel, Sharma, Maya, & Kielhorn, 2014; Lynch, 1995; Mimura, 1995; Niklas et al., 2005; Schachtman, Reid, & Ayling, 1998). However, our results also show that seedling P content scales isometrically with N content across the four species, whereas prior studies report that a ⅔ scaling exponent for P versus N across large plants (Han, Fang, Guo, & Zhang, 2005; Niklas, 2006; Niklas et al., 2005; Reich et al., 2010; Wright et al., 2004). This difference may be partly due to the gradual decrease in physiologically active biomass (and the N contained therein) as plants continue to grow in size (Ågren, 2008; Niklas & Enquist, 2002; Peng et al., 2010). As seedlings lack “necromass” compared to their larger woody counterparts, the scaling exponents for P versus N are expected to be higher, which helps to explain why the numerical value of the scaling exponent for P versus N changes as a function of plant ontogeny.

## 5 CONCLUSIONS

We have shown that the scaling relationships for seedling respiration with respect to whole‐plant N and P content numerically differ between angiosperm and gymnosperm species, and that they also differ between the growing and dormant season. It follows therefore that there can be no “canonical” (invariant) scaling relationship for the effects of N and P content on respiration, which helps to explain different results reported by a variety of researchers. Although no canonical scaling relationship exist, it is clear that patterns emerge when the scaling relationships among N, P, and respiration are tracked over the course of plant ontogeny, as, for example, the decline in respiration rates with increasing plant biomass. Further research is clearly required to gain deeper insights into this phenomenology because statistical correlations among these variables of interest do not in and of themselves provide for mechanistic explanation.

## CONFLICT OF INTEREST

The authors declare they have no conflicts of interest.

## AUTHOR CONTRIBUTIONS

RRF, DLC, and QLZ conceived and designed the experiments. RRF, ML, and YZ performed the experiments. RRF, JS, and FCY analyzed the data. RRF and DLC wrote the manuscript.
