# Supplementary material for: Divergent scaling of respiration rates to nitrogen and phosphorus across four woody seedlings between different growing seasons
Source: Ecol Evol. 2017 Sep 18;7(21):8761–9. doi: 10.1002/ece3.3419 (PMC5677492; doi:10.1002/ece3.3419)
Supplement: Supplementary file 1 [file ECE3-7-8761-s001.docx]

**APPENDIX S1** The basic morphometrics of four evergreen woody species (mean ± *SE*)

| *Species* | Functional  types | Life  form | Family | Basal Diameter  (cm) | Height  (cm) |
| --- | --- | --- | --- | --- | --- |
| *C. lanceolata* | Gymnospermae | Needle | Taxodiaceae | 4.81±0.11 a | 34.43±0.73 a |
| *P. massoniana* | Gymnospermae | Needle | Pinaceae | 3.52±0.09 b | 30.00±0.70 b |
| *M. Pauhoi* *^ | Angiospermae | Broad-leaved | Lauraceae | 3.54±0.11 b | 32.67±1.10 a |
| *P. Bournei* *^ | Angiospermae | Broad-leaved | Lauraceae | 3.01±0.09 c | 34.65±1.08 a |

Shade tolerant species indicated by *; Species only found in China were indicated by ^;

Different letters in a column indicated that significant differences between species at *p* < 0.05.
